# Clinical electromagnetic brain scanner

**DOI:** 10.1038/s41598-024-55360-7

**Published:** 2024-03-08

**Authors:** Amin Abbosh, Konstanty Bialkowski, Lei Guo, Ahmed Al-Saffar, Ali Zamani, Adnan Trakic, Aida Brankovic, Alina Bialkowski, Guohun Zhu, David Cook, Stuart Crozier

**Affiliations:** 1 School of Electrical Engineering and Computer Science, The University of Queensland, St Lucia, QLD4072 Australia; 2 Faculty of Medicine, The University of Queensland, St Lucia, QLD4072 Australia

**Keywords:** Biomedical engineering, Electrical and electronic engineering, Translational research, Stroke

## Abstract

Stroke is a leading cause of death and disability worldwide, and early diagnosis and prompt medical intervention are thus crucial. Frequent monitoring of stroke patients is also essential to assess treatment efficacy and detect complications earlier. While computed tomography (CT) and magnetic resonance imaging (MRI) are commonly used for stroke diagnosis, they cannot be easily used onsite, nor for frequent monitoring purposes. To meet those requirements, an electromagnetic imaging (EMI) device, which is portable, non-invasive, and non-ionizing, has been developed. It uses a headset with an antenna array that irradiates the head with a safe low-frequency EM field and captures scattered fields to map the brain using a complementary set of physics-based and data-driven algorithms, enabling quasi-real-time detection, two-dimensional localization, and classification of strokes. This study reports clinical findings from the first time the device was used on stroke patients. The clinical results on 50 patients indicate achieving an overall accuracy of 98% in classification and 80% in two-dimensional quadrant localization. With its lightweight design and potential for use by a single para-medical staff at the point of care, the device can be used in intensive care units, emergency departments, and by paramedics for onsite diagnosis.

## Introduction

Each biological tissue has unique dielectric properties that vary with frequency^1^. Any change in the physiological and pathological conditions of affected tissues will have a corresponding change in dielectric properties, and thus the response of affected tissues to EM waves will change. For example, A hemorrhagic stroke, which involves bleeding in the brain, increases the dielectric properties (permittivity and conductivity) of tissue, while a clot, which blocks blood flow, reduces those properties compared to the healthy tissue. Thereby, proper sensors can, in principle, be designed and used to illuminate those tissues using specific signals and capture scattered signals that carry the required information to detect, image, and monitor those changes. EMI has been widely investigated to build medical diagnostic tools that are portable, safe, affordable, and non-ionizing. In the specific application of brain imaging, EMI is targeted to play a critical role as an onsite, real-time, or quasi-real-time detection and classification tool. An EMI device is comprised of two main elements. The hardware platform includes an array of EM transceivers to efficiently illuminate the brain with EM pulses and to capture scattered waves. The software element of the device includes special-purpose algorithms to process the collected data and generate an image of the brain showing any stroke. The physics behind EMI is the contrast in the dielectric properties between healthy and stroke-affected tissues^2^.

While EMI techniques have been demonstrated in laboratory settings^3–5^, they have faced serious challenges that hindered their adoption for clinical use. Those challenges are associated with the hardware, such as the number, type, and location of sensors (antennas), used coupling/decoupling media between those sensors and head, significant noise and external clutter/interference, which can be stronger than stroke response to EM waves, frequency band, reliable system calibration, software (pre- and post-processing algorithms), computational time and so on. The most challenging aspect of EMI is the requirement to detect extremely weak stroke signals (much less than  − 50 dBm for deep targets)^6^ in the presence of strong signals from the surrounding tissues and structures.

Many techniques have been formulated to enable EMI methods in non-invasive assessment, and monitoring of the functional and pathological conditions of tissues. Those techniques can be classified as radar or tomography techniques^5^; although statistical and artificial intelligence methods are also emerging^7,8^. Radar techniques, which require a wide frequency band to achieve acceptable image resolution^9^, use back-projection to locate abnormal scattering objects, such as strokes, within the imaged domain^10,11^. To find those targets, radar methods assume that the EM wave propagates in straight lines in each medium between the transmitter to the receiver, but this is a rough approximation to a complicated scenario. The transmitting antenna will most likely operate in its near field and does not emit a well-defined beam, but rather an EM field that propagates in different directions due to the heterogeneous nature of the brain and multiple propagation pathways through reflections and diffractions^12,13^. EM waves in the frequency range of interest, have a spherical wavefront. They are initiated at a point in space and sent in all directions. This is vastly different from frequencies in the visible light and X-ray spectrums, which propagate in effectively a single direction only. Through careful design of the antenna elements, the spherical wavefront can be limited to a uni-directional radiation pattern^14^. In head imaging, the reflected signals from the skin and skull are much stronger than those reflected from the stroke. In fact, the stroke response is embedded in skin reflections^15^. Thus, pre-processing techniques, such as sensors’ rotation and various average-trace signal subtractions including entropy-based methods, are utilized to eliminate strong reflected signals^16^. The optimization techniques and software-derived templates are also used to accurately detect the location of the target^11^. Also, in EMI, a matching medium fills the gap between EM sensors and the imaged object and reduces skin interface reflections, like using gel in ultrasound^17^.

EM tomography (EMT) is another technique utilized to map the dielectric properties of the brain^18^. It processes the captured scattered fields from the brain using computationally intensive iterative algorithms^19^. However, due to the limited number of available EM antenna sensors due to limited space around the head, the relatively large size of those sensors, and many unknowns in the brain map, EMT is typically an ill-posed problem^20,21^. Thus, optimization techniques are usually utilized to obtain the solution. Since the governing equations of EMT are nonlinear, those optimization methods are thus nonlinear, taking significant time and computational resources to solve^22^. Approximations to linearize those equations can be used; for example, assuming the antennas as point sources is used to reduce the computational time at the expense of solution accuracy^23^. Despite all those techniques, EMT may not lead to a unique solution due to the intrinsic non-uniqueness from the evanescent EM waves and diffraction, local minima traps, and sensitivity of the EMT inverse problem to noise^24^. Moreover, it is always a challenge to create a simulation environment that accurately emulates the experimental situation. With the limited available data and thus ill-posed situation, any minor difference between the two environments might lead to incorrect imaging results^25^. Those challenges, especially the ill-posed situation, sensitivity to noise, and long computational time, face the other approach to tomography, i.e. Electrical Impedance Tomography, which uses signals at the kHz frequency range. While some promising imaging results have been achieved, mostly in simulations^26,27^, EIT is yet to be proven as a reliable clinical imaging method.

Various types of EMI systems for brain imaging have been designed, proposed, and implemented using one of the aforementioned techniques with promising results. However, those systems face challenges when being translated into clinical use^28^. For example, the device in^29^ uses a statistical classification algorithm that relies on machine learning^30^ from a large data library to indicate the type of stroke. While that solution shows potential in triage decision support with its stroke detection algorithm, its reported specificity is still low and does not generate an image. On the other hand, the system reported in^31^ is complex, having 177 antennas and a complicated switching matrix, and may require significant computational resources. The recent results from a modified system, which has 192 antennas, indicate its clinical potential^32^.

## Methods

**Figure 1:**
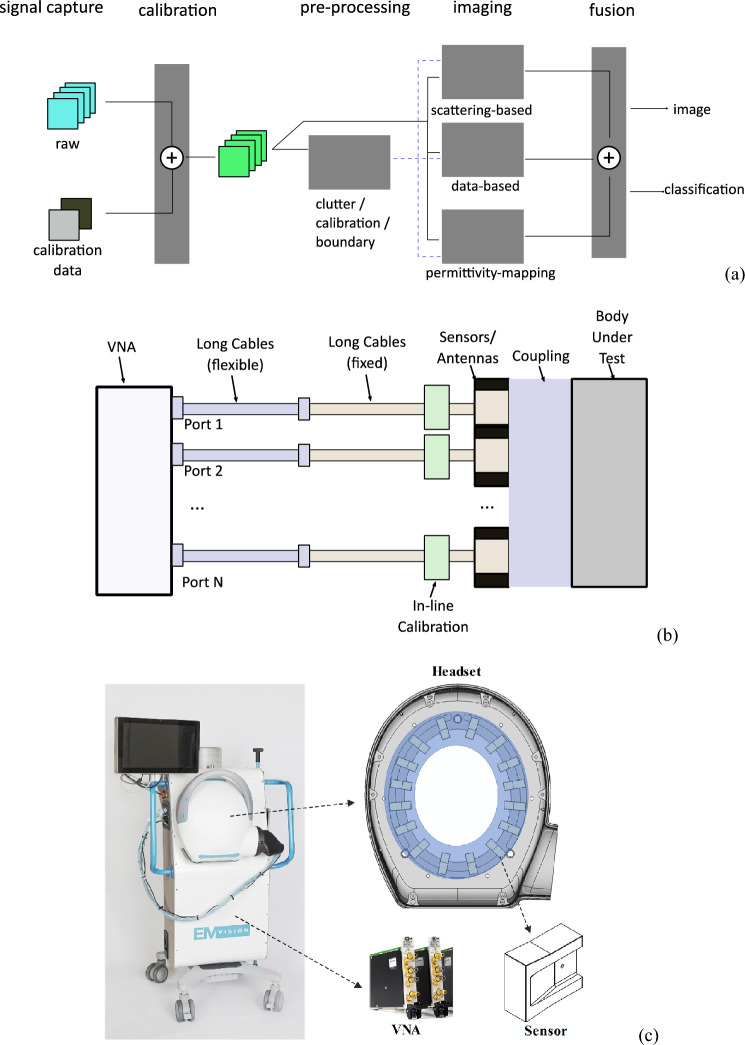
(**a**) Framework of the device: In the first step, the system is calibrated using well-defined phantoms. The system is then used to scan the patient’s head and capture scattered signals, which are calibrated against calibration phantom signals. The calibrated data is then processed using a neural network to estimate the contour of the head slice being scanned. Those boundaries are then fed to line crossing, direct mapping, and beamography, which work in an iterative collaborative manner to detect, localize and classify any stroke. In parallel, a tripartite data-driven algorithm is used to map the dielectric properties of all brain tissues. Finally, all the generated images from those algorithms are fed to a fusion algorithm, to give the final image, which shows the location, size, and shape of the stroke in addition to the internal tissue distribution. (**b**) Main elements of the device: A coupling medium between the antenna array and the head, an antenna array surrounding the head and operating at the low microwave frequency band, an in-line calibration unit for each antenna element, two sets of fixed and flexible cables connecting the antenna array to the multi-port vector network analyzer. (**c**) The developed device that meets the clinical requirements.

The head has a complex dielectric profile with several layers including skin, skull, fat, cerebral spinal fluid (CSF), grey matter, and white matter. Each head tissue has dispersive properties and distinct relative permittivity and conductivity^33^. Tissues surrounding the grey and white matter are small in volume and propagation length but provide a large contrast. The skull has quite a low permittivity, and the CSF has an exceptionally large permittivity. The heterogeneous structure of the head and the wide range of values for the dielectric properties of the brain create a difficult EM propagation environment, characterized by multipath propagation and interference. Thus, the adopted approach in the reported device is a complementary combination of novel physics-based and data-driven techniques to enable the utilization of their strengths and alleviate their weaknesses for a final accurate EM image. A flow chart of the adopted framework is shown in Fig. 1a. In the first step, the system is calibrated using well-defined phantoms^25^. The system is then used to scan the patient’s head and capture scattered signals, which are calibrated against calibration phantom signals. The calibrated data is then processed using a neural network to estimate the contour of the head slice being scanned^34,35^. Those boundaries are then fed to line crossing^36^, direct mapping^37^, and beamography^38^, which work in an iterative collaborative manner to detect, localize and classify any stroke. In parallel, a tripartite data-driven algorithm^39^ is used to map the dielectric properties of all brain tissues. Finally, all the generated images from those algorithms are fed to a fusion algorithm, to give the final image, which shows the location, size, and shape of the stroke in addition to the internal tissue distribution.

The frequency range for the reported device is selected to balance the requirements of theoretical resolution and penetration of EM waves into the head. Higher frequencies would provide higher resolution but would have limited penetration into the head. Most of the signal would be reflected and absorbed by the skin and skull layers of the head. Lower frequencies have higher penetration but also require larger antenna structures and provide lower resolution. The size of the antenna also dictates the number of antenna elements that can fit around the head. The richness of the information provided to an EMI device is improved with more frequency bandwidth, more antenna elements, and antennas that allow signals to penetrate over the entire head. Based on these needs, the reported device operates in the frequency range of 0.7–1.8 GHz and has 16 antenna elements.

A Keysight PXI Vector Network Analyser (VNA) is used as a 16-port transceiver for data acquisition. The VNA excites the transmitting antennas serially and captures signals received by all antennas at each excitation. The dynamic range of the system is selected to ensure that the system can capture both the strong signal reflected off the boundary of the head as well as the much weaker signal reflected off anomalies inside the head. As the ratio of these two values does not change with increasing transmitted power, that power is maximized while ensuring the subject’s safety as per relevant guidelines^40^ and keeping the system-level noise to a minimum. This ensures that the lowest level signal with information from inside the head is above the thermal noise level.

### Prototype EMI

A schematic diagram detailing the main hardware components of the device and how they interface with one another can be seen in Fig. 1b, whereas a photo of the device is shown in Fig. 1c. Each of the main elements is discussed in the following paragraphs.

#### Coupling medium

It is a mixture of materials selected to ensure antenna-head matching and thus maximum EM penetration into the head. The medium has a dielectric constant of 45 and a conductivity of 0.15 S/m at 1 GHz^25^. It is contained within a 1 mm thick silicone membrane and fills the void between the antennas and the head. It is designed to have a minimal loss to ensure that the signal is not attenuated significantly before entering the head. A fluidic system is utilized to maintain a consistent pressure of the coupling media inside the membrane throughout imaging, consisting of a peristaltic pump, pressure sensor, and pressure relief valve. This was also used to retract the membrane to allow the patient to be properly positioned within the system before inflation.

To fill the region between the head and antennas’ apertures with coupling liquid, a thin silicone membrane is attached above and below those antennas at the edges of the decoupling region to allow the entire area within the main beam of the waveguide antennas to be filled with the coupling liquid while keeping the liquid separate from the head. Using this membrane ensures that the patient does not endure discomfort from being in contact with the liquid, which should not be contaminated by the patient or environment, allowing it to be reused for the shelf life of the coupling medium. The used membrane has a thickness of 0.5 mm, and thus has negligible impact on signal penetration, appearing similar to the patient’s hair.

A peristaltic pump, housed within the trolley, is used to transport the liquid coupling medium from the reservoir (also housed within the trolley) to the membrane. Thin PVC tubing, which is connected to the headset inlet and outlet, is used to link the membrane and the reservoir. These tubes are also enclosed in the harness containing flexible RF cables. A digital pressure gauge monitors the pressure being applied to the head and provides feedback to the pump so that the desired pressure is maintained. Using this system, reliable data is enabled by conforming the membrane to the patient’s head shape as much as possible. The maximum pressure is 0.5 PSI when measuring to ensure the patient’s comfort, and so fail-safes are built into the system so that this is not exceeded while on a patient.

#### Decoupling medium

To further improve the signal information from the head, a lossy medium surrounding the antennas prevents any stray signals from directly traveling between antennas, or further distorting useful signals via multiple reflections. This decoupling layer is designed using a polymer composition with a permittivity close to 45, and a significant loss (conductivity of 4.5 S/m at 1 GHz) to ensure that signals entering this medium can be ignored.

#### Absorber

External EM interference, generated by other wireless devices, such as mobile phones, operating at the same frequency band, is a major problem for EMI. Thus, in addition to the decoupling medium, EM shielding techniques or absorbers are used to mitigate any interference^41^. A metal-backed EM absorber is utilized in the reported device to provide shielding from outside signals and absorb any internal stray signals. The absorber consists of a multilayer of magnetic substrates and a single-layer lossy frequency selective surface^42^. A single-square loop geometry is used for the lossy surface, which is fabricated using the resistive sheet. The magnetic layers are made by mixing different ratios of carbonyl-iron powder with a silicone elastomer called polydimethylsiloxane. The absorber provides  − 15 dB reflectivity over the entire used bandwidth.

#### EM sensor antennas

Due to the complexity of the human head, the device uses a combination of different algorithms, which require the antenna to (a) operate in a wide range of frequencies and pulse durations, (b) have a constant phase center across the operating frequencies, (c) be compact such that more antenna elements can be placed around the head to obtain more information, and (d) provide directional radiation patterns to concentrate the transmitted waves towards the head. All the above requirements make the antenna design challenging.

The antenna design is shown in Fig. 1c. It is based on a waveguide-type radiator. It has a rectangular aperture, while the rest of the antenna surface is covered by conductive paint. To reduce the operating frequency to start from 0.65 GHz as needed for head imaging while keeping the antenna compact, it is filled with loss-less high-permittivity ceramic to ensure a high radiation efficiency. The wideband operation is achieved by modifying the shape of the waveguide. The feeding section, i.e., the part of the waveguide near the feeding position, works as a double-ridge waveguide to reduce the operating frequency for antenna miniaturization purposes. A tapered transition from the ridge waveguide to the rectangular aperture is used to improve impedance matching. Also, by lengthening the height, the antenna provides a more focused beam in the Z direction, which helps avoid clutter from above and below the array.

#### System calibration

EMI in the microwave regime is strongly influenced by the stability of the measurements. This necessitates effective calibration schemes to eliminate undesirable variations due to the environment. Thus, an in-line calibration method is devised to ensure the accuracy and repeatability of measurement data. Before deploying the system in a clinical environment, a full system calibration is performed. During the collection of each set of measurement data in the clinical environment, the in-line calibration circuits are used to attach a series of measurement standards to the port at the plane of the antennas. Data collected from these standards is used to move the measurement plane to the input of the antennas and refresh the calibration for each measurement using a modified calibration technique. This was especially necessary to combat the effects of cable movement within the system, an inherent challenge when the headset needs to be independent of the transceiver.

Each of the sensor antennas is connected directly to the in-line calibration circuit, to enable software-controlled calibration of the system when collecting data in the clinical environment. The states of these switches are controllable using a custom ARM-based microprocessor board, connected to the main trolley computer over USB. The in-line calibration circuits are connected directly to an SMA aggregating interface panel on the headset via semi-flexible coaxial cables of varying lengths. The headset is then connected back to another interface on the side of the trolley through flexible 1.5 m long coaxial cables, which are then connected to the ports of the VNA inside the trolley.

Another significant challenge is presented by the potential for patient movement throughout the measurement data collection window. Movements of the patient within the imaging domain can cause significant variations and instability in the measurements and can also cause cable calibration data to become ineffective if the headset moves notably beyond the position in which it was characterized before measurement. To mitigate this, the in-line calibration circuits are connected to a single standard and continuously monitored before beginning measurements when placed on a patient. If the level of change in consecutive measurements is above the signal-to-noise ratio required to generate accurate images, the system continues to wait until the patient reaches a stable state, after which measurement data is collected in a few seconds.

Two homogeneous calibration phantoms are fabricated to have properties above and below the average dielectric properties of the head. These phantoms serve as reference object that has well-known and deliberately chosen properties. By acquiring the scattering parameters for the two calibration phantoms, along with the scattering parameters for the patient’s head, a range of calibration schemes can be performed by algorithms to counter certain types of faults in measurements, eliminate the impact of variations of environments, manufacturing imperfections or even changes in properties of the device with time^25^.

**Figure 2:**
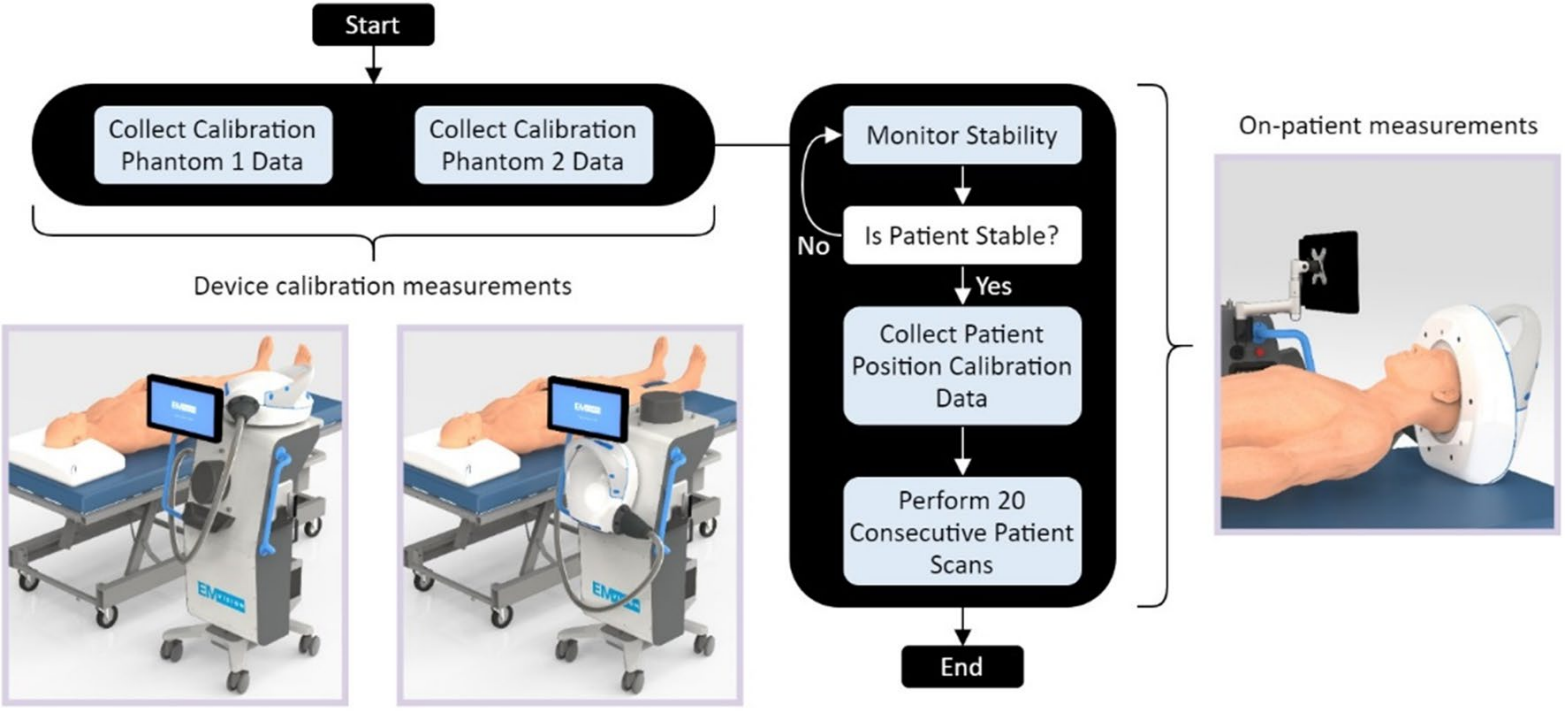
Device initialization and scanning. Before a clinical scan, a full 16-port calibration is performed. Each of the antennas is connected directly to the in-line calibration circuit, to enable software-controlled calibration of the system when collecting clinical data. The in-line calibration circuits are connected directly to the aggregating interface panel on the headset via semi-flexible coaxial cables. The headset is then connected back to another interface on the side of the trolley through flexible cables, which are then connected to the VNA. To mitigate the impact of a patient’s head movement, the in-line calibration circuits are connected to a single standard and continuously monitored before beginning measurements when placed on a patient. If the level of change in consecutive measurements is above the signal-to-noise ratio required to generate accurate images, the system continues to wait until the patient reaches a stable state, after which measurement data is collected in a few seconds. Two homogeneous calibration phantoms serve as reference object that has well-known and deliberately chosen properties. By acquiring the scattering parameters for the two calibration phantoms, along with the scattering parameters for the patient’s head, a range of calibration schemes can be performed by algorithms to counter certain types of faults in measurements, eliminate the impact of variations of environments, manufacturing imperfections or even changes in properties of the device itself with time.

The main steps to initialize the device before scanning a patient are shown in Fig. 2

### Software

After capturing the data, and calibrating it using the two reference phantoms, the calibrated data is then fed to the following series of algorithms designed to achieve specific objectives.

Algorithm-I: Boundary detection- Incorporating boundaries of the imaging object as a priori information to imaging algorithms can significantly improve the performance of EMI systems. To avoid overly complicating the device by using additional sensors, a multi-layer deep neural network method is used to estimate the boundary (external contour) of the imaged object using the calibrated EM data^35^. Reflection coefficients are useful parameters for boundary detection algorithms based on machine learning. The model enables fast scan and image creation while eliminating the need for additional devices in accurate boundary estimation.

**Figure 3:**
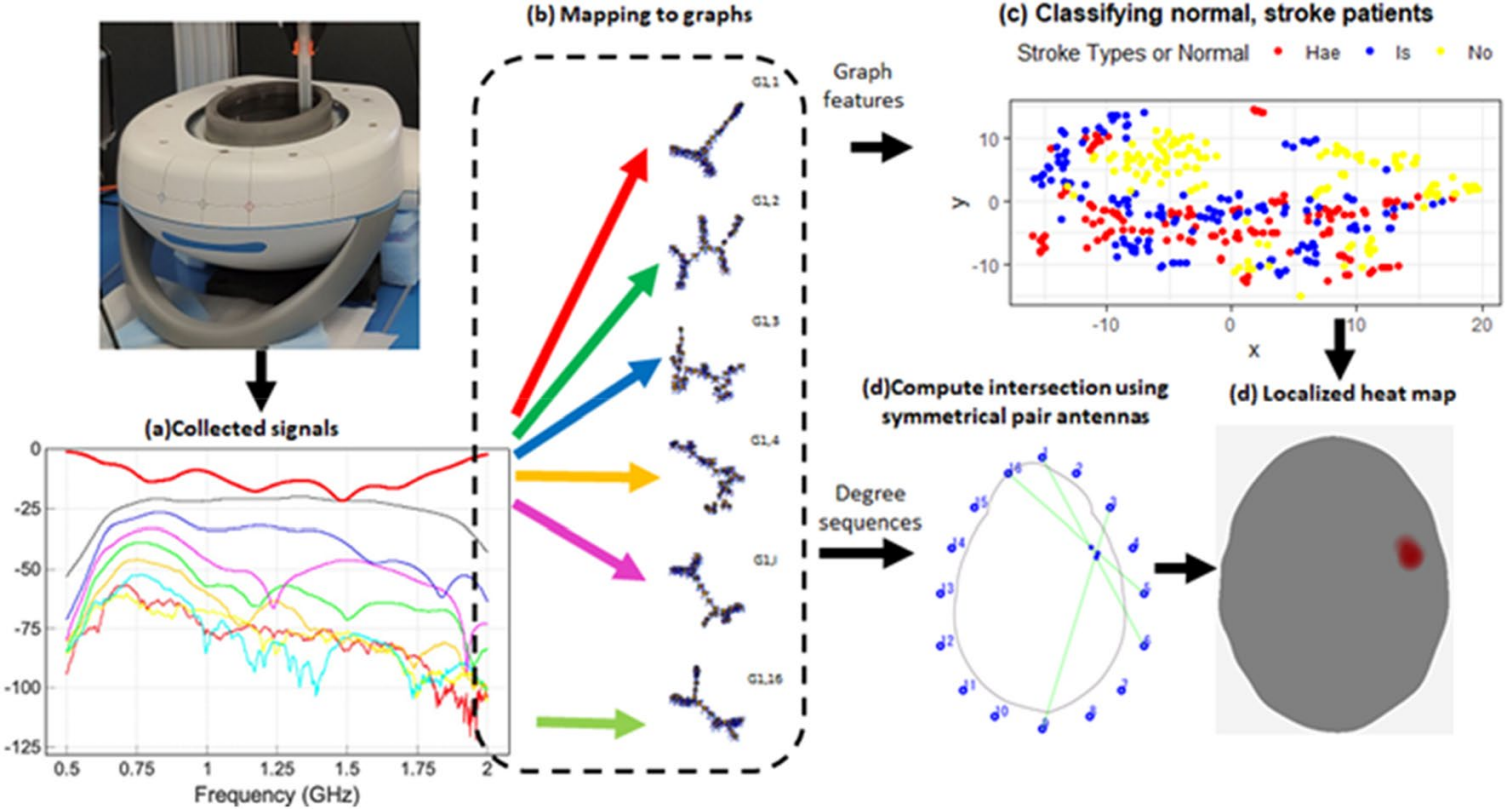
Algorithm-II: It converts the calibrated time-domain data into a horizontal visibility graph based on a linear fast-weighted horizontal visibility graph algorithm. For classification, three similarity matrices of degree, strength, and entropy are constructed and used to form three differential matrices between left and right brain hemispheres to distinguish healthy/unhealthy brains and classify stroke if unhealthy. Using extracted features from these matrices, four different classifiers (Random Forest, K-near neighbor, naïve Bayes, and support vector machine) are used to form probability classifying results. Healthy brains are assumed to be approximately symmetric, and strokes will lead to significantly different amplitude and phase changes compared to healthy brains. By taking pairs of symmetrically located antennas around the head, a differential graph degree (or entropy) is calculated. Lines crossing strokes have significantly larger entropy than those not crossing a stroke. Visualization of the line-crossing data is implemented through training.

Algorithm-II (Fig. 3): Mixed graph similarity and line crossing are used to detect, localize, and classify strokes. Both algorithms first convert the calibrated time-domain data into a horizontal visibility graph based on a linear fast-weighted horizontal visibility graph algorithm.

For classification, three similarity matrices of degree, strength, and entropy are constructed and used to form three differential matrices between left and right brain hemispheres to distinguish healthy/unhealthy brains and classify stroke if unhealthy. Using extracted features from these matrices, four different classifiers are used: Random Forest^43^, K-near neighbor^44^, naïve Bayes^45^, and support vector machine^46^. The classification outputs include distinguishing between healthy and potential hemorrhagic (ICH) and ischemic (IS) strokes, based on the current data available. The number of classes is not limited by the algorithm but rather by available datasets of conditions that also exhibit changes in permittivity and conductivity. Probability classifying results are generated by combining those methods. For localization, the generated visibility graphs within the detected head boundaries (from pre-processing) are used. Healthy brains are assumed to be approximately symmetric, and strokes will lead to significantly different amplitude and phase changes compared to healthy brains. By taking pairs of symmetrically located antennas around the head, a differential graph degree (or entropy) is calculated. Lines crossing locations where strokes are located will have significantly larger entropy than those not crossing a stroke^47^. Together, these two linked algorithms provide the location and classification of the strokes, knowing that they are trained on the twenty scans of each of the first 12 patients from the clinical trial.

**Figure 4:**
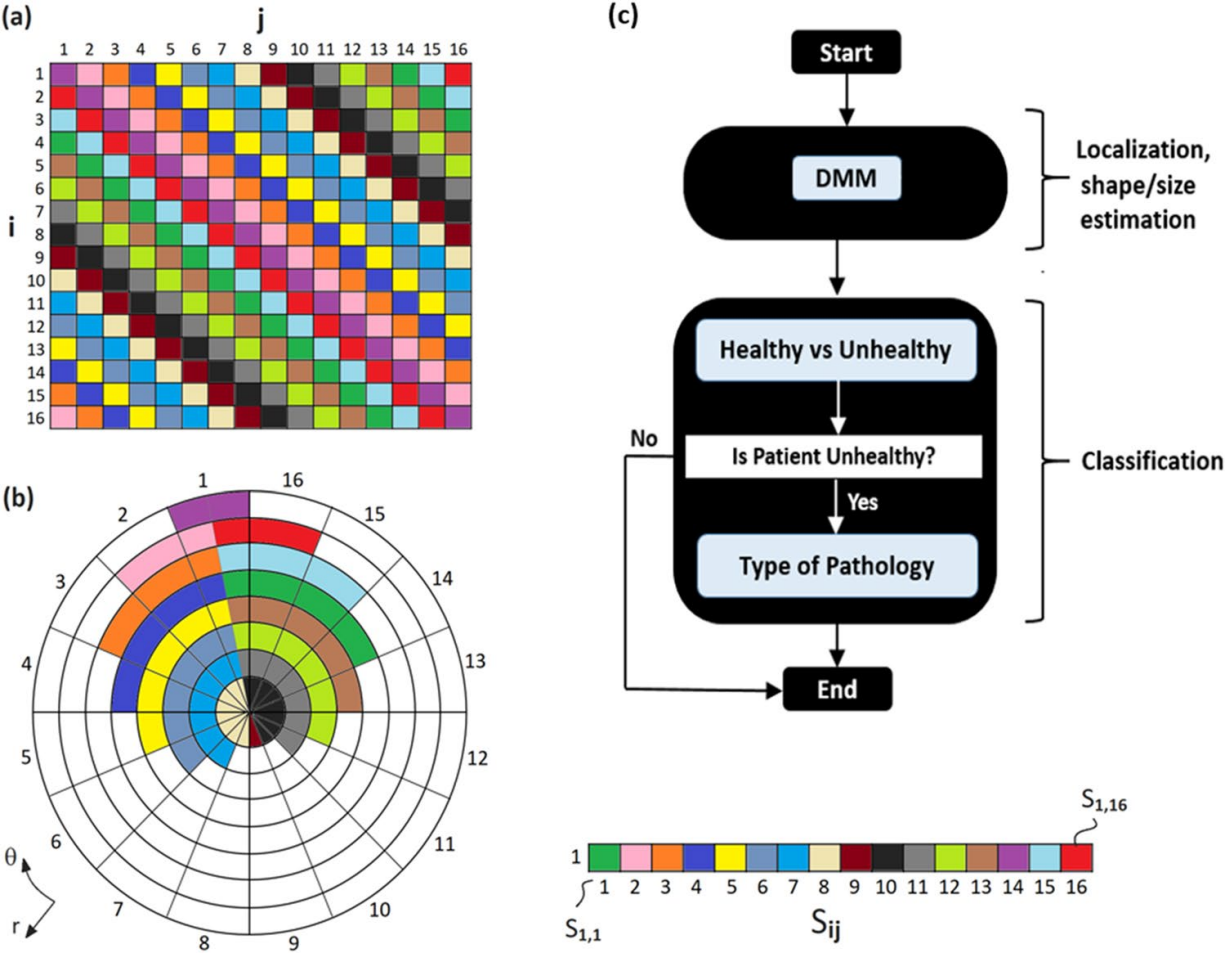
Algorithm III: Receive calibrated data as a matrix (in a 16 × 16 Cartesian grid) to generate a polar grid with a unique parameter mapping scheme as shown by individually color-coded cells. The included subplot shows one example of the encoding scheme for antenna no.1, and this scheme is repeated for all other antennas in succession. Another subplot shows the DMM flowchart wherein localization is performed first, followed by classification of the patient in terms of ‘*healthy vs unhealthy’* and *‘ischaemic vs hemorrhagic’* stroke type if *‘unhealthy’* is detected. Classification solvers also employ the DMM core algorithm as part of their kernel, along with statistical calculations to estimate the likelihood levels of classified types.

Algorithm-III (Fig. 4): At its core, the Direct Mapping Method (DMM) provides an elegant approach for re-mapping the calibrated EM signals to a polar coordinate system, which is more congruent with the anatomical shape of the human brain. As shown in Fig. 4, for each antenna, this mapping process is guided by a unique S-parameter map, which maps a pair of transmitting and receiving antenna to a location in the polar coordinate system. It follows the principle that antennas close to each other are sensitive to shallow anomalies, whereas antennas far away from each other are sensitive to deeper anomalies. The mapping system is augmented by a receiver sensitivity profile, which is specific to each antenna array. DMM can be applied for all the collected frequency points individually and then aggregated to yield the image output. For classification purposes, the DMM solver is used by establishing thresholds based on two statistical calculations to determine: (a) using the overall intensity of the output, if a stroke is present or not (i.e., healthy patient) and (b) using the phase information of the output, what type of stroke is present (i.e., ischaemic, or hemorrhagic). For pre-processing, different variations of the background field mitigation can be a uniform background reference and/or brain symmetry (i.e., brain self-reference), which ultimately tends to provide superior stroke localization and classification results.

**Figure 5:**
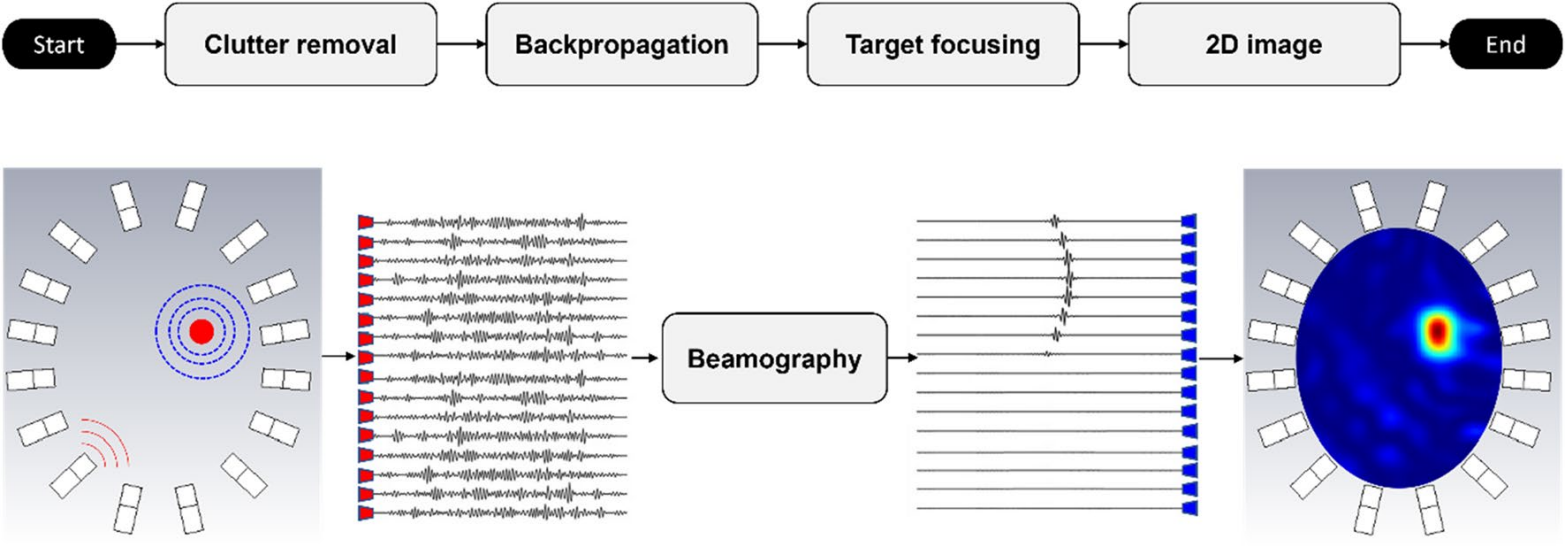
Flowchart of beamography, and an example. Beamography detects and localizes strokes by performing two tasks (1) clutter mitigation, and (2) target focusing. Clutter mitigation is accomplished through symmetry and average subtractions. Marginal distortions due to the inevitable anatomical brain asymmetry and the head displacement are alleviated by average subtraction, where the average of all signals at all frequency samples is subtracted from each of them. After clutter removal, the data captured by each sensor is back-propagated to the imaging domain, using the domain Green’s function to calculate the field observed at each point in the brain for each transmitter–receiver pair and each frequency sample. Those calculated fields are then superposed to construct the final image. The location of the stroke in this method is shown by a higher intensity compared to healthy tissues.

Algorithm-IV (Fig. 5): Beamography, i.e. beamforming plus tomography, detects and localizes strokes within the brain by performing two major tasks 1) clutter mitigation, and 2) target focusing. Clutter mitigation is accomplished through symmetry and average subtractions to suppress the clutter returns from other tissues than the stroke. Clutter mitigation in EMI means removing the effect of healthy tissues inside the imaging domain. The effect of signals from healthy tissues is estimated using brain symmetry. The head is largely symmetrical across the sagittal plane and when a unilateral lesion, such as a hemorrhage or ischemia, occurs, it distorts that symmetry. Hence, signals from the healthy side can be used as a reference to extract the stroke’s response. Knowing the affected side of the brain, which is provided using the distance correlation function^48^, the signals of the healthy side, including the signals that pass the central line, are subtracted from the unhealthy side signals to get stroke signals. Marginal distortions due to the inevitable anatomical asymmetry of the brain and the head displacement inside the brain are then alleviated by average subtraction, where the average of all signals at all frequency samples is subtracted from each of them. After signal clutter removal, the data captured by each sensor is back-propagated to the imaging domain, using the imaging domain Green’s function^49^ to calculate the field observed at each point in the brain for each transmitter–receiver pair and each frequency sample. Those calculated fields are then superposed to construct the final image. The location of the stroke in this method is shown by a higher intensity compared to healthy tissues.

**Figure 6:**
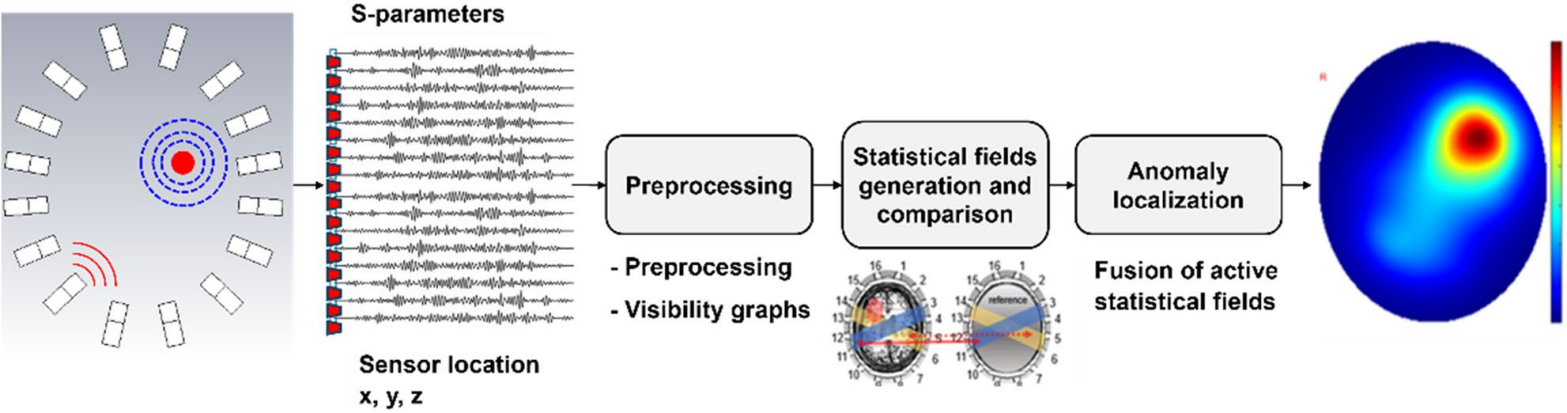
Flowchart of stroke localization with EVSLA algorithm. The algorithm takes S parameters of the raw measurement and calibration data in the frequency domain and locations of sensors as inputs. In the preprocessing step, the algorithm performs inverse Fourier transform (IFFT) of collected S parameters and generates visibility graphs. Generated graphs are used to construct statistical fields and compute the similarity levels between the calibration and raw data. Regions with higher dissimilarity are kept active and used for fusion. The obtained fusion matrix represents the likelihood of each pixel being affected by a stroke. Visualization of the fusion matrix shows the stroke location.

Algorithm-V (Fig. 6): Unsupervised ML algorithm (Expected Value-based Stroke Localization (EVSLA)) is based on a statistical comparison of symmetrical brain patches and takes advantage of a highly symmetrical human brain^50^. To localize the anomaly in the brain, the algorithm creates pairs of symmetric patches that have the form of a 4-sided polygon. Signals collected from pairs of antennas bounding symmetric patches are represented as a complex network and statistically compared to generate so-called statistical fields. The information contained in individual statistical fields is fused by discretizing the imaging domain into pixels and computing the expected value of statistical fields for every pixel individually. The resulting expected value matrix is visualized such that pixels with higher intensity show a higher expectancy of an anomaly being present.

**Figure 7:**
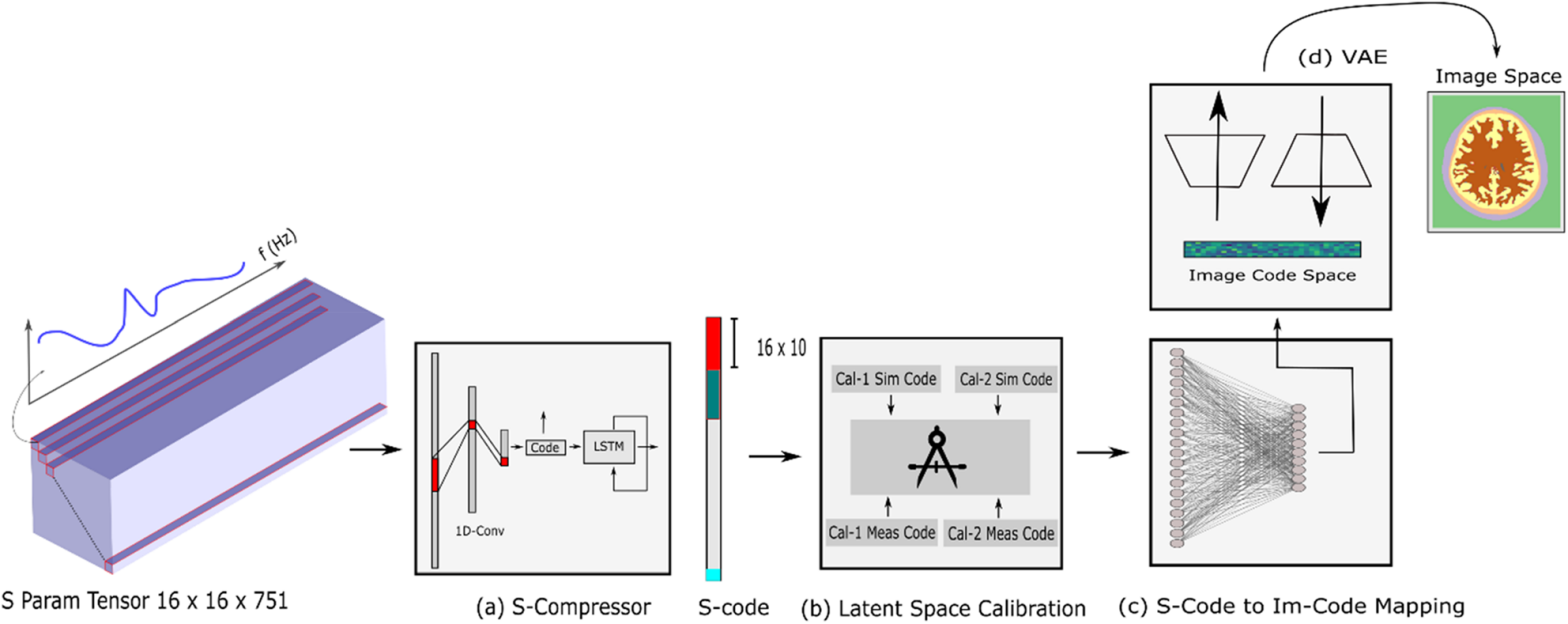
DeepHead for dielectric mapping of the brain. It uses double compression to maximally utilize the cheap unlabelled data to provide a priori information required to ease under-determinism and reduce the sensitivity of inference to the input. The developed neural network model is tripartite, two of its ends are compressors that learn manifolds of interest in both input (captured data by the sensors) and output side (image) of the world. The first compressor uses large datasets of sensor data measured on a physical array. The data here does not need to be labeled. The second compressor uses simulated data with known permittivity distributions. A third module learns to connect the two earlier independently trained compressors using cheap unlabelled data. Lastly, a latent space calibration concept is introduced to enable the work on real-life data as the supervised training of the third module is performed on simulation data. The result is a stable solver with a high-resolution output.

Algorithm-VI (Fig. 7): EMI medical imaging in the microwave regime is a hard problem notorious for instability and under-determinism. This two-pronged problem is tackled using dielectric mapping via DeepHead algorithm as a new imaging paradigm. It is a two-pronged solution that uses double compression to maximally utilize the cheap unlabelled data to a) provide a priori information required to ease under-determinism and b) reduce the sensitivity of inference to the input. The presented permittivity mapping algorithm is a data-based approach that uses calibrated signals over a range of frequencies to provide a map of permittivity inside the imaging domain at a specific frequency. Only limited data is available to train a network using electromagnetic signals to a map of permittivity hence requiring a unique neural network structure.

The developed neural network model is tripartite, two of its ends are compressors that learn manifolds of interest in both the input (captured data by the sensors) and the output side (image) of the world. The first compressor uses large datasets of sensor data measured on a physical array. The data here does not need to be labeled. The second compressor uses simulated data with known permittivity distributions. The simulated data involves segmented heads synthesized from an MRI with 1 mm × 1 mm × 1 mm resolution. Various strokes representing the dielectric properties of hemorrhagic and ischemic are inserted into those models, which include the main tissues (skin, skull, fat, cerebellum, white matter, gray matter, and CSF) with their dispersive dielectric properties. The head models are automatically placed into random locations within the imaging domain of the Computer Simulation Technology (CST) environment to emulate the clinical scenario.

A third module learns to connect the two earlier independently trained compressors using cheap unlabelled data. Lastly, a latent space calibration concept is introduced to enable the work on real-life data as the supervised training of the third module is performed on simulation data. The result is a stable solver with a high-resolution output. The technique is a fully data-driven implementation of the paradigm proposed in the context of microwave brain imaging. It infers the dielectric distribution of the brain at a desired single frequency while making use of an input that spreads over a wide band of frequencies.

**Figure 8:**
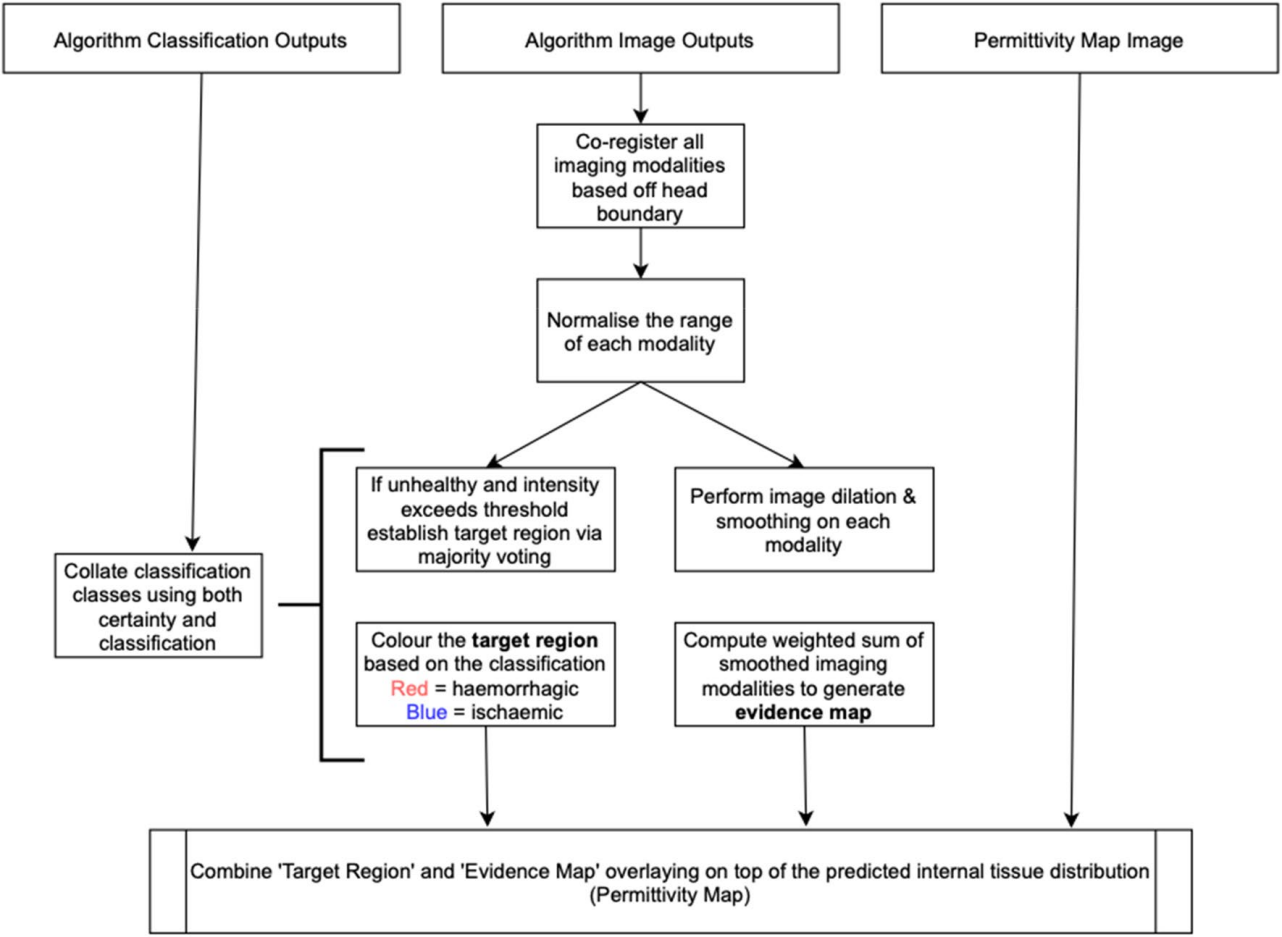
Image fusion is used to combine the complementary information from different algorithms and generate the final image using the following steps: Co-registering different modalities into the same co-ordinates using an elastic warping algorithm, normalizing each modality to have the same intensity scale, finding the region of agreement between the algorithms by applying a threshold to the normalized images and returning the region of agreement based on the overlap. Each modality is then smoothed, and an image dilation operation is performed to produce closed-in and smooth target detection, and the region of agreement is superimposed on the smoothed modality outputs to generate the agreed target location as well as additional heatmap information provided from the different modalities. The final target location and heatmap are then colored based on stroke classification and superimposed on the neural network-based permittivity mapping output image.

Algorithm-VI (Fig. 8): Image Fusion is used to combine the complementary information from different imaging algorithms, some of which are physics-based whereas others are data-driven, to generate a single combined output image. This consists of the following steps: Co-registering the different modalities into the same coordinates using an elastic warping algorithm to normalize all modalities to the same domain and normalizing each modality to have the same intensity scale. After co-registration and normalization, the region of agreement between the algorithms is determined, by applying a threshold to the normalized images and returning the region of agreement based on the overlap. Each modality is then smoothed, and an image dilation operation is performed to produce closed-in and smooth target detection, and the region of agreement is superimposed on the smoothed modality outputs to generate the agreed target location as well as additional heatmap information provided from the different modalities. The fusion method is also used to decide the final classification by combining the decisions of individual classifiers and certainty levels in a multi-classifier system. Thus, the assignments from different classifiers are fused to utilize the strengths of different classifiers and alleviate any weaknesses or limitations into one final decision. The final target location and heatmap are then colored based on the stroke classification (red for hemorrhagic and blue for ischemic) and superimposed on the neural network-based permittivity mapping output image.

## Results

A single-site study was conducted at the Princess Alexandra Hospital (PAH) in Brisbane, on 50 patients with diagnosed ischaemic or hemorrhagic stroke. Patients who are in a conscious condition were scanned using the reported device within the time window from a few hours up to 5 days from the stroke onset. The time of the EM scan is ensured to be as close as possible to the conventional imaging modalities (e.g., MRI or CT) without interrupting the patient’s treatment. The EM data collection procedure includes performing 20 consecutive scans for each patient (each scan taking about 1.5 s) to maximize the available time window for capturing patient data and ensure we have enough data for each patient and that any outlier can be removed before processing (e.g. patient movement during a scan). The study includes subjects with clinically and/or radiologically established strokes. Subjects with a history of previous cerebrovascular events, i.e., old strokes were not excluded from the study. The study and all relevant experimental protocols were performed following the relevant guidelines and regulations and were approved by the Human Research Ethics Committee, Metro South Health, Queensland, Australia (HREC/2019/QMS/48520). The subjects included in the study were chosen thoughtfully to identify the capabilities of the system to detect the target within and out of the field, deep and shallow, to detect and localize both types of strokes as well as to test the capabilities of the algorithm.

The primary goal of this study was collecting data for training algorithms, testing usability, and verifying safety. The study also verified the capability of the developed device in clinical settings compared to “ground truth” CT and/or MRI scans. The provided CT and MRI images in this study serve as a relative ground truth (GT) since the CT images show a set of 1 mm thick slices of the head, while the EMI device scans ~ 60 mm thick slice of the head (equal to the height of the sensor). Thus, the available MRI and CT scans can serve just as a relative GT. Furthermore, feedback was collected from clinicians and patients on the usability and comfort of the prototype. No intervention or modification to the standard of care of hospital-based treatment of stroke was done as part of this study. The study enrolled and processed datasets from 50 patients (37 ischaemic and 13 hemorrhagic) representing the diversity of stroke in localization, size, and clinical severity. The mean age was 67.7 years of age with the majority, 72% of patients, aged 60 years and over. There were equal numbers of male and female patients. Of the 50 patients, 29, (58%) had only a CT performed whereas 21, (42%) had CT/MRI performed. Patients were scanned with a non-contrast CT in the first step to exclude the presence of hemorrhage. The presence of stroke symptoms with an absence of hemorrhage supports the likelihood of an ischemic event. Depending on initial images obtained with CT, patients were then scanned with perfusion CT and/or MRI. As a result of these scans, 26% of patients were diagnosed as having had a hemorrhagic stroke and 74% as having had an ischaemic stroke. The National Institutes of Health Stroke Scale (NIHSS) was used to measure stroke severity and was recorded for 49 patients. The mean NIHSS score was calculated as 5.9 which indicates moderate severity. The participating patients’ de-identified CT and/or MRI ground truth scans were interpreted and classified independently by radiologists. After the datasets were processed by the algorithm team, the algorithm classification and localization outputs were verified by clinical investigators and advisors.

Inclusion Criteria: Adults >  = 18 years of age, who are admitted to hospital with new neurological signs and confirmed diagnosis of stroke supported by conventional brain imaging. Participants should be able to provide written informed consent with the ability to adhere to the study visit schedule and other protocol requirements. The selected participants should have confirmed diagnosis of stroke within 72 h of admission with a head size deemed suitable for scanning with the current brain scanner.

Exclusion Criteria: Experiences seizures from the onset of stroke or known history of seizure episodes; Has an injury or known medical condition on the head that would not allow the placement of EMI brain scanner; Is unable to lie still for the duration of the scan; Is not a suitable candidate according to the assessing investigator; Has any metal implants in the head or neck, for example, stents, aneurysm clips, surgical clips, pressure monitors and drains; and is known to be pregnant or lactating.

Study Procedure: Potential participants with a confirmed diagnosis of stroke are reviewed to participate in the study. The participant was assessed and, if eligible, the participant or the participant’s legal representative was approached for consent to participate in the study. Informed consent was thus obtained from all participants included in the study. After consent, the first scan using EMI brain scanner is conducted and follow-up scans are conducted as deemed appropriate by the investigator. Each scan is repeated to obtain paired image acquisitions for comparison. Patients are followed for up to 28 days following admission as inpatients or until discharge (whichever is sooner).

**Figure 9:**
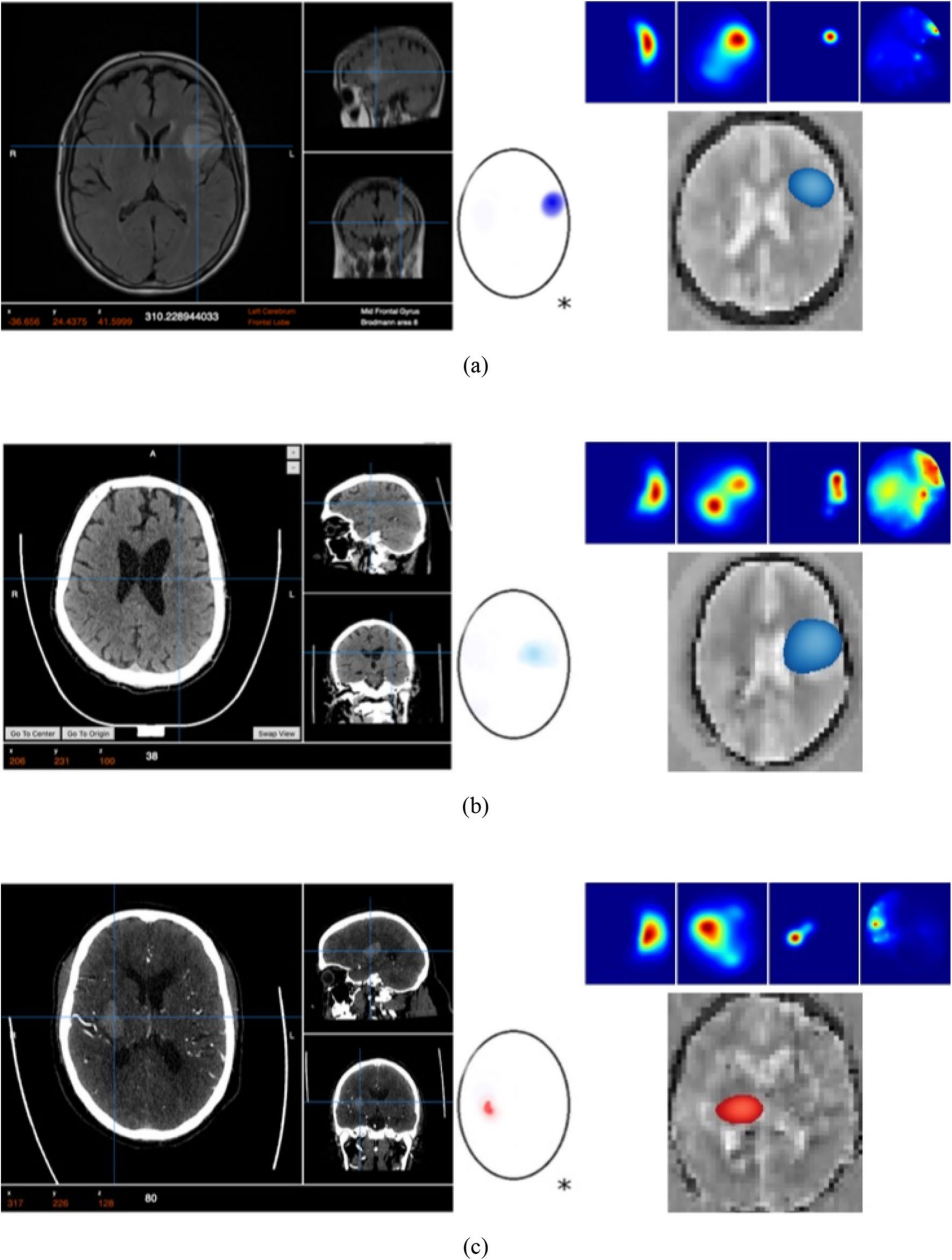

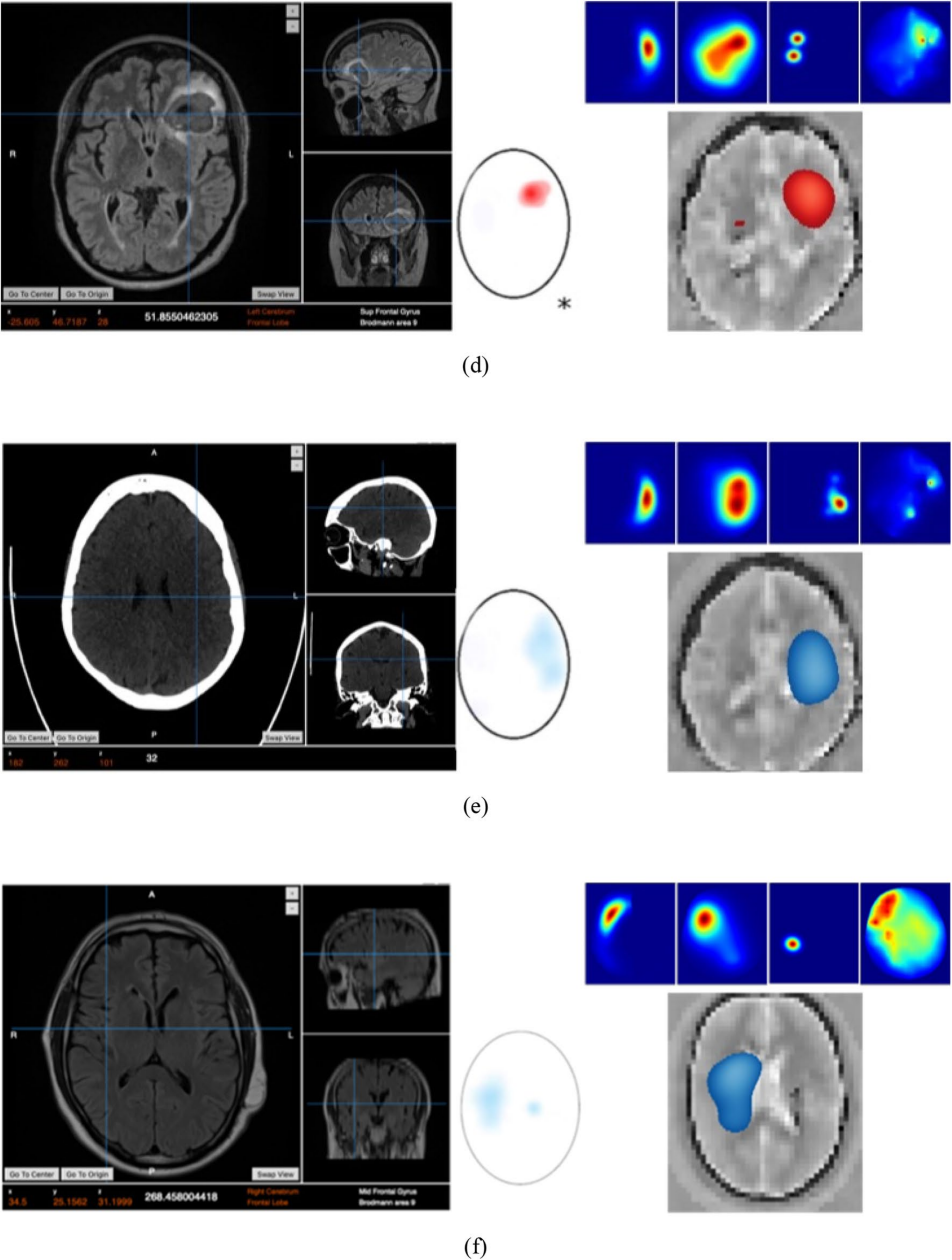

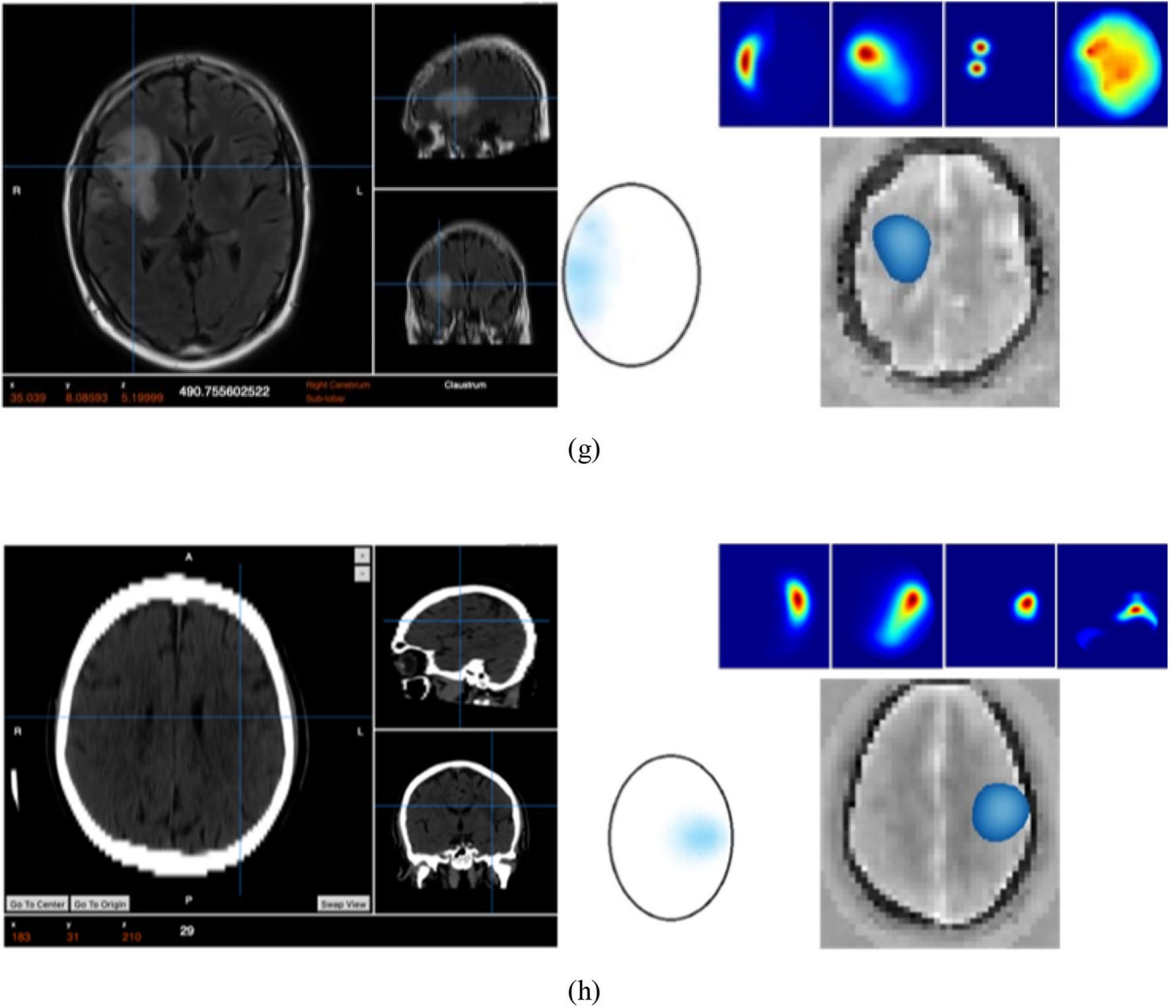
Patient data from clinical trial presenting the ground truth on the left side, a set of outputs from the four individual algorithms (right side–top row), the clinically interpreted ground truth, and the fused output, which shows the detected area colored in red for hemorrhagic stroke and blue for ischemic stroke.

The results from using EMI for the 50 cases are analyzed relevant to the ground truth that is produced through conventional medical imaging. A representative set of 7 cases is presented in Fig. 9. In the presented images, each case is presented as a set of ground truth and interpreted ground truth images on the left side, and EM images on the right side. In the EM images, a colormap represents the stroke probability level, which indicates the probability of an area being affected by the stroke. Compared to the GT, the constructed EM images demonstrate that the utilized device can detect and localize both types of strokes in the correct quadrant of the head, which is a good indicator to clinicians of the stroke presence and location.

The evaluation of localization is based on whether the fusion images resulted in target detection in the same quadrant as the ground truth scans (CT/MRI). For any scenarios where the ground truth image or fusion image had multiple areas of pathology identified, the clinical verifier has taken the most prominent/intense area to be the area of interest. The fusion images were observed to be able to localize in the correct quadrant with an overall accuracy of 80% using this metric. The device classification was observed to demonstrate an ability to differentiate between hemorrhagic and ischaemic stroke with an overall accuracy of 98% in the full sample (50).

Secondary endpoints for the operator and patient feedback were reported by a Likert scale (1–5), with a score of 1 representing ‘strongly agree’ and 5 representing ‘strongly disagree’ on the ease of use and comfort to patients. Despite being a prototype, the device was well tolerated by both operators (1.6–1.9/5) on the ease of use, and patients scoring (1.2–1.4/5) on the comfort and ease of scanning. The feedback was consistently positive. Furthermore, there were no device-related adverse events reported for the patients defined in the primary study analysis.

## Discussion

**Figure 10:**
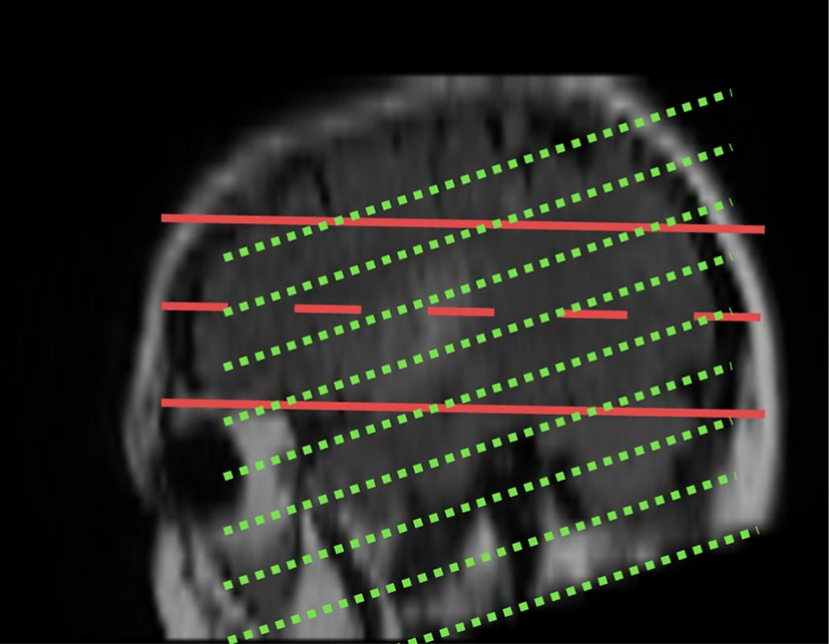
Sagittal plane of the human head showing the plane of imaging in EM and MRI slices. The plane of the original MRI slices is depicted with green dotted lines, and the two solid red lines show the imaging volume of the array. The dotted red line indicates the axial center of the antenna array.

The results confirm that the device is successful in detecting, localizing, and classifying strokes as well as providing an indication of stroke size. The current prototype and algorithms relied on an array of antennas in a single plane and produced a 2D image depicting the imaged volume. The plane of the antenna array was designed to capture most of the brain volume, by making the center line angled from a position above the eyes and covering the back of the head. It is important to take this rotation into account when comparing slices of existing medical imaging modality output to the produced output. In Fig. 9, all the images in the ground truth images have been rotated to ensure that the axial view presented represents the plane of the EM imaging. To understand this rotation better, in Fig. 10, the sagittal (side) view is used to illustrate the differences. The plane of the original MRI slices is depicted with green dotted lines, and the red dotted line shows the imaging volume of the array. As the MRI includes a set of voxels, the volume can be rotated to ensure that a meaningful comparison can be made.

The best localization performance is obtained when the anomaly is within the volume in front of the antenna array, which is marked with the two solid red lines in Fig. 10. Although reduced imaging performance for strokes outside this range, the device was still able to classify the anomaly correctly. This indicates that the presence of stroke has a more global effect than just the core of stroke.

Though the presented results are promising, there are several potential improvements on the current device that should be addressed in the future. The image generation is 2D because the current system only has one layer of sensors, even though it covers most of the brain volume. This limitation can be addressed if the data is collected using a three-dimensional sensor array or the current array is moved to alternate positions and rescanned for complete brain coverage. Another aspect to be investigated in future studies is the capability of the algorithm to detect small targets (less than a few mL). The detection of stroke using the current system is that of the affected area in the imaging volume, rather than simply the detection of the core of the stroke as can be interpreted from existing imaging modalities. For example, a 5 × 5 mm area defining the core of a stroke might only depict a small fraction of the volume affected by the stroke, as EMI perceives tissues differently from MRI and CT. The detection of smaller strokes located deeply in the brain where the target response might be embedded in noise and hence harder to differentiate, would be the subject of future investigations.

From the computational perspective, the execution time of the reported device on each patient depends on the utilized computing tool. In the presented study, the utilized processing software is executed on an Intel(R) Xeon(R) W-2145 machine, with a 3.7 GHz CPU, 64 GB of RAM, and a 64-bit Operating System with an average time of 1 min, which makes it suitable for real-time prehospital use in emergency stroke scenarios.

## Data Availability

The data that support the findings of this study were processed by the University of Queensland research team and are available from the data owners EMVision Medical Devices, but restrictions apply to the availability of these data, which were used under license for the current study, and so are not publicly available. Data are however available from the co-author, K. Bialkowski, upon reasonable request conditional on the permission of the owner of the data, EMVision Medical Devices.
